# The Amuvatinib Derivative, *N*-(2H-1,3-Benzodioxol-5-yl)-4-{thieno[3,2-d]pyrimidin-4-yl}piperazine-1-carboxamide, Inhibits Mitochondria and Kills Tumor Cells under Glucose Starvation

**DOI:** 10.3390/ijms21031041

**Published:** 2020-02-04

**Authors:** Ran Marciano, Hila Ben David, Barak Akabayov, Barak Rotblat

**Affiliations:** 1 Department of Life Sciences, Ben-Gurion University of the Negev, Beer Sheva 8410501, Israel; ranmarci@post.bgu.ac.il (R.M.); hilabend@post.bgu.ac.il (H.B.D.); 2 The National Institute for Biotechnology in the Negev, Ben-Gurion University of the Negev, Beer Sheva 8410501, Israel; 3 Department of Chemistry, Ben-Gurion University of the Negev, Beer-Sheva 8410501, Israel; akabayov@bgu.ac.il

**Keywords:** medicinal chemistry, cancer metabolism, amuvatinib, mechanistic target of rapamycin (mTOR), synthetic lethality

## Abstract

Glucose levels inside solid tumors are low as compared with normal surrounding tissue, forcing tumor cells to reprogram their metabolism to adapt to such low glucose conditions. Unlike normal tissue, tumor cells experience glucose starvation, making the targeting of pathways supporting survival during glucose starvation an interesting therapeutic strategy in oncology. Using high-throughput screening, we previously identified small molecules that selectively kill cells exposed to glucose starvation. One of the identified compounds was the kinase inhibitor amuvatinib. To identify new molecules with potential antineoplastic activity, we procured 12 amuvatinib derivatives and tested their selective toxicity towards glucose-starved tumor cells. One of the amuvatinib derivatives, *N*-(2H-1,3-benzodioxol-5-yl)-4-{thieno[3,2-d]pyrimidin-4-yl}piperazine-1-carboxamide, termed compound 6, was found to be efficacious in tumor cells experiencing glucose starvation. In line with the known dependence of glucose-starved cells on the mitochondria, compound 6 inhibits mitochondrial membrane potential. These findings support the concept that tumor cells are dependent on mitochondria under glucose starvation, and bring forth compound 6 as a new molecule with potential antitumor activity for the treatment of glucose-starved tumors.

## 1. Introduction

Cells within solid tumors are often starved for glucose [1,2]. This is due to increased glucose uptake and utilization on the one hand [3,4] and on the other, a reduced nutrient supply due to defective vasculature [5]. How tumor cells adapt to such conditions is an important question as normal tissue does not suffer from glucose starvation and may not be as dependent on these pathways. Thus, these adaptive pathways offer a therapeutic window [6].

Several cellular pathways supporting adaptation to glucose starvation have been identified, including the unfolded protein response [7], fatty acid beta-oxidation [8], the mammalian target of rapamycin (mTOR) [9], adenosine monophosphate (AMP)-activated protein kinase (AMPK) [10,11] and the p63-SOX2 axis [12]. In addition to their function in these pathways, the mitochondria were found to play an important role in cell adaptation to glucose starvation, leading to the idea that mitochondria could be targeted in cancer [1]. 

To identify new compounds to selectively kill glucose-starved tumor cells, we performed a high throughput screen in glucose-starved cells and controls and identified 31 such compounds, two of which, papaverin and avastin, were found to inhibit mitochondria in culture and synergize with antiangiogenics in vivo [13].

One of our positive hits was amuvatinib, a kinase inhibitor, known to inhibit cell growth and increase the sensitivity of tumor cells to chemotherapy and radiotherapy. Other studies showed that amuvatinib inhibited DNA repair and protein synthesis [14,15,16]. Whether amuvatinib is toxic upon glucose starvation is not known, nor is it known if amuvatinib has specific structural features promoting toxicity under glucose starvation. To increase the portfolio of compounds selectively killing tumor cells under glucose starvation, we procured 12 amuvatinib derivatives and tested their activity in culture. One of these compounds, *N*-(2H-1,3-benzodioxol-5-yl)-4-{thieno[3,2-d]pyrimidin-4-yl}piperazine-1-carboxamide (compound 6), was found to be more potent than amuvatinib in a cell line specific manner, reducing mitochondrial membrane potential and the mTOR pathway under glucose starvation. Our data suggest that compound 6 is a new molecule with potential anti-cancer activity, and support the notion that glucose-starved cells are dependent on the mitochondria.

## 2. Results

**Figure 1:**
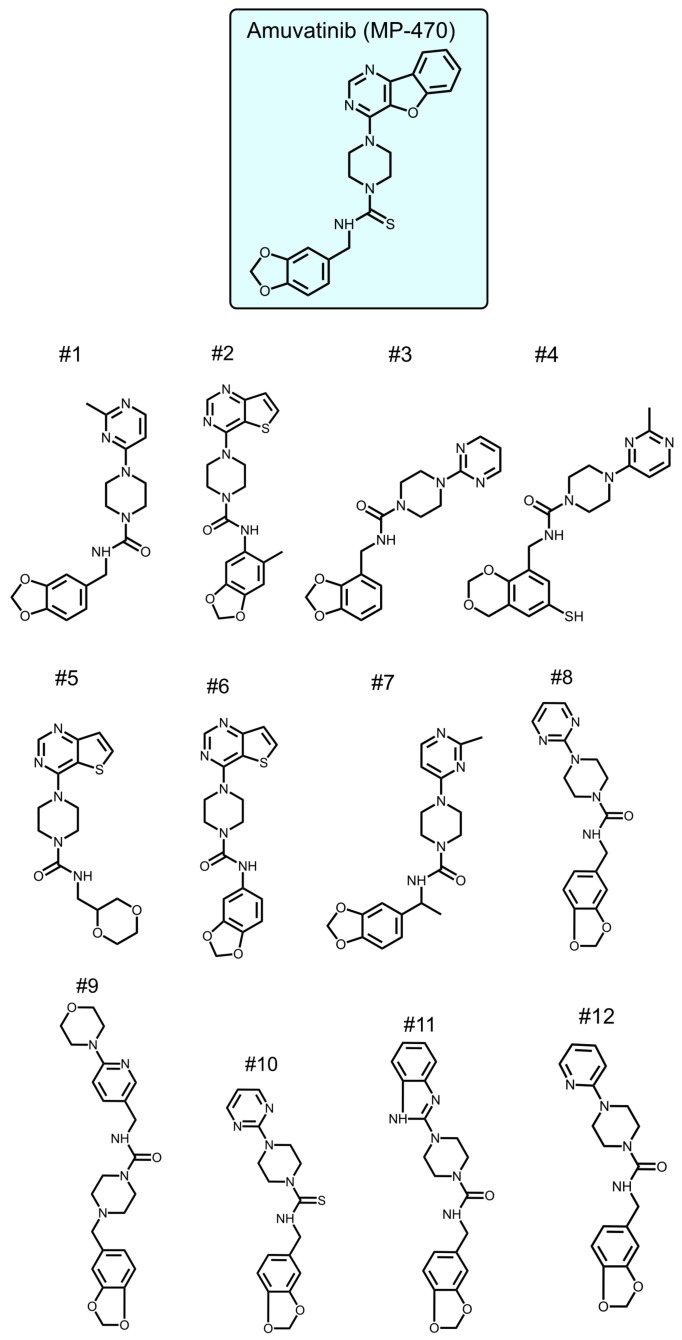
Amuvatinib derivatives.

**Figure 2:**
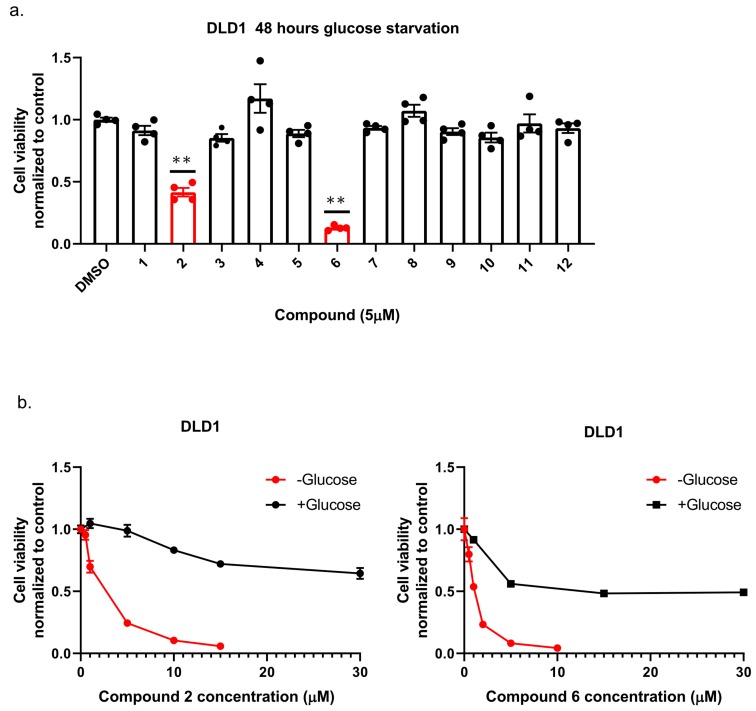
The selective toxicity of 12 amuvatinib derivatives under glucose starvation. (**a**) Bar graph showing relative cell viability of DLD1 tumor cell line treated with 5 µM of compounds 1–12 for 48 h under glucose starvation was determined using Crystal Violet staining. Cell viability is relative to the control group (equal volume of dimethyl sulfoxide (DMSO)). Bars of compounds 2 and 6 are colored in red. ** *p* < 0.0001. (**b**) Kill curves of compounds 2 and 6 with and without glucose. Relative cell viability was measured using Crystal Violet after 48 h with and without glucose.

To identify new compounds with selective toxicity towards glucose-starved tumor cells, we procured 12 amuvatinib derivatives (Figure 1). To test their biological activity, we first used the same pipeline used in our previous study [13]; namely, we plated colon cancer DLD1 tumor cells in glucose-free medium supplemented with 5 µM of each compound for 48 h, after which we measured cell viability using Crystal Violet staining (Figure 2a). We found that the compounds: 1-Piperazinecarboxamide, N-(6-methyl-1,3-benzodioxol-5-yl)-4-thieno[3,2-d]pyrimidin-4-yl (cas# 1957378-39-0) and 1-Piperazinecarboxamide, N-(1,3-benzodioxol-4-ylmethyl)-4-thieno[3,2-d]pyrimidin-4-yl (cas# 1954932-35-4) (hereafter, compound 2 and compound 6, respectively), were toxic upon glucose starvation as compared with controls. To confirm these findings, we performed similar experiments using different concentrations of compound 2 and compound 6. We found glucose starvation enhanced the toxicity of both compounds, and that compound 6 exhibits stronger toxicity (Figure 2b). 

**Figure 3:**
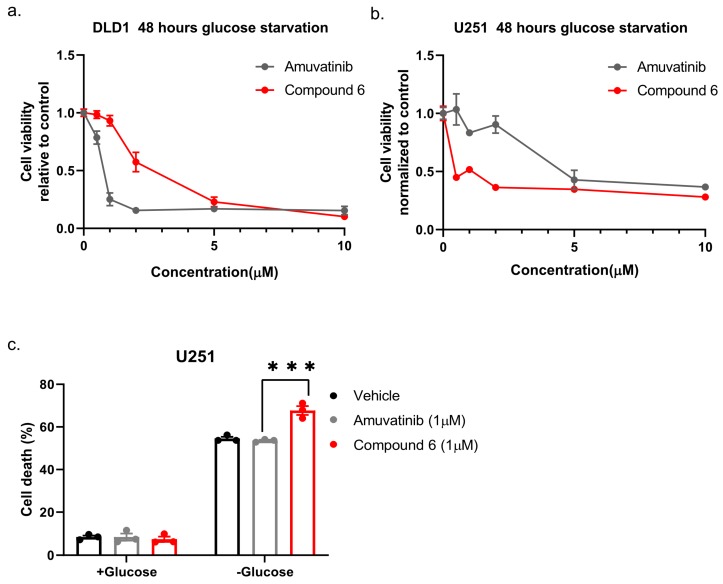
Comparing the potency of compound 6 to amuvatinib. (**a**) Cell viability of DLD1 or U251 (right) treated with the indicated compounds for 48 h: amuvatinib (gray line) or compound 6 (red line). (**b**) Cell death of U251 Cells treated with the indicated compounds for 16 h in glucose-starved medium. (**c**) Cell death was measured by propidium iodide (PI) staining and fluorescence-activated cell sorting (FACS). Cells were treated with 1 µM of either Amuvatinib (grey bars) or compound 6 (red bars). Vehicle (black bars): the same volume of DMSO as in the highest concentration of compound 6. *** *p* < 0.0001.

Next, we compared compound 6 to the mother molecule, amuvatinib. We treated DLD1 or U251 cells with glucose starvation and of escalating dose of compound 6 or amuvatinib for 48 h after which we measured viability using Crystal Violet staining. While amuvatinib was more toxic towards glucose-starved DLD1 cells, compound 6 was more toxic towards glucose-starved U251 cells (Figure 3a), by comparison. To test if the observed reduction in viability was due to increased cell death, we plated U251 cells in normal or glucose-free media in the presence of amuvatinib or compound 6 for 16 h, after which cell death was measured using propidium iodide (PI) staining and fluorescence-activated cell sorting (FACS) (Figure 3b). In agreement with the viability assay, cell death was increased in the glucose-starved cells treated with compound 6 as compared to amuvatinib, while both compounds were significantly more toxic under glucose starvation as compared with controls. Together, these data indicate that compound 6 is more potent than amuvatinib under glucose starvation in a cell line dependent context. 

**Figure 4:**
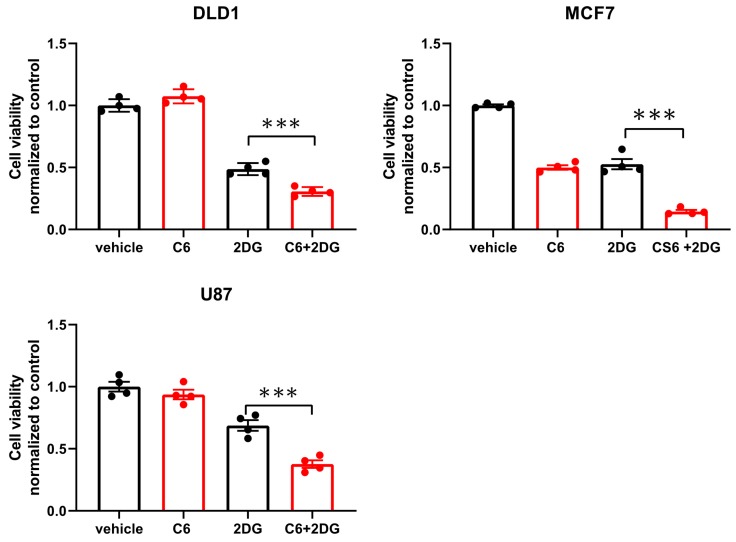
Compound 6 is toxic under glycolysis inhibition. Relative cell viability of the indicated cell lines treated for 24 h with: vehicle (the same volume of DMSO as in the highest concentration of compound 6 with glucose) and 5 µM of compound 6 (C6), 2DG (25 mM), alone or in combination. Cell viability was measured using Crystal Violet staining. Results are normalized to control (vehicle). *** *p* < 0.0001.

While glucose starvation is a physiological condition existing within solid tumors, we wanted to test if compound 6 was also toxic in other forms of energetic stress. To this end, we used the glycolysis inhibitor, 2-deoxy-glucose (2DG), to induce energetic stress. DLD1 cells were treated with compound 6 (5 µM) and a high concentration of 2DG (25 mM) alone or in combination for 24 h, after which viability was measured using Crystal Violet staining (Figure 4). 2DG treatment led to reduced viability, which was enhanced in the presence of compound 6, while compound 6 on its own did not lead to reduced viability. We confirmed these findings using breast and brain cancer cell lines, MCF7 and U87, respectively (Figure 4).

**Figure 5:**
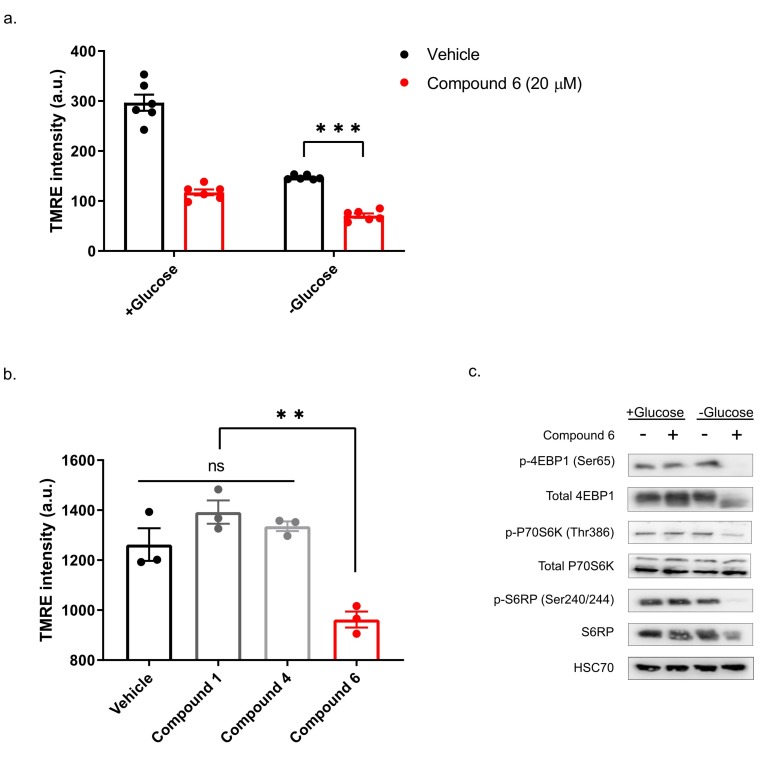
Compound 6 significantly reduced mitochondrial membrane potential. (**a**) The mitochondrial activity in DLD1 cells was measured by tetramethylrhodamine, ethyl ester (TMRE) and FACS. DLD1 cells were treated with compound 6 (20 µM) (red), or vehicle (black) (the same volume of DMSO) for three hours with and without glucose. a.u. =arbitrary units. *** *p* <0.0001. (**b**) Mitochondrial activity of DLD1 cells treated with vehicle (black) or 10 µM of compounds 1, 4 (grey) and compound 6 (red) for 3 h. ** *p* < 0.01. (**c**) Inhibition of the mTOR pathway in U251 cells treated with compound 6 (10 µM) for 3 h in the presence or absence of glucose, after which cell lysates were collected and analyzed by immunoblot using the indicated antibodies.

Our previous study showed that compounds exhibiting selective toxicity upon glucose starvation have two features: they inhibit the mTOR pathway upon glucose starvation; and they inhibit mitochondrial membrane potential independently of glucose starvation [13]. To test if compound 6 exhibits these features, we treated DLD1 cells with glucose-free or normal media and 20 µM compound 6 or vehicle for three hours, after which we measured mitochondrial membrane potential, using tetramethylrhodamine, ethyl ester (TMRE) and FACS (Figure 5a). Compound 6 significantly reduced mitochondrial membrane potential in glucose proficient or deficient conditions, suggesting that compound 6 is a mitochondrial toxin. To further validate this conclusion, we treated U251 cells with compounds #1 or #4 that are not toxic under glucose starvation, or compound 6, for 3 h, after which we measured mitochondrial membrane potential using TMRE and FACS (Figure 5b). In agreement with our model, only compound 6 significantly reduced mitochondrial membrane potential.

Finally, we asked if compound 6 inhibits the mTOR pathway upon glucose starvation. We treated U251 cells with compound 6 (10 µM) and glucose starvation for 3 h, after which we lysed the cells and compared the activity of the mTOR pathway with untreated cells, using western blot (Figure 5c). We found a marked reduction in phosoho-4EBP1, phospho-S6kinase and phospho-S6 in cells treated with a combination of compound 6 and glucose starvation, but not in cells treated with either one of these stresses alone. Together, these data indicate that compound 6 treatment leads to inhibition of the mTOR pathway only in glucose-starved cells, as was the case with other compounds identified previously to selectively kill tumor cells under glucose starvation [13].

## 3. Discussion

Tumor cells depend on the functions of the electron transport chain for survival under limited glucose [1]. In accordance, we previously identified several mitochondrial toxins exhibiting selective toxicity upon glucose starvation [13]. Here, we asked if compound 6—a derivative of one of our positive hits which exhibits selective toxicity upon glucose starvation—is inhibiting mitochondrial membrane potential, and found that this is indeed the case.

How compound 6 inhibits mitochondrial membrane potential is not known. Comparing the structure of the two active compounds to that of the non-active ones shows that the two active molecules share similar molecular structure, containing three functional chemical groups: 1,3-benzodioxol, thieno[3,2-d]pyrimidine, and piperazine. The relatively high mass of both molecules covers a larger chemical space with the potential to yield a similar stable network of weak interactions with the potential protein target. We do not know what this target might be, but we speculate that it is a protein involved in the electron transport chain.

The distinct metabolic state of tumor cells, known as the Warburg effect, is characterized by increased glycolysis as compared with oxidative phosphorylation [17]. Increased glycolysis supports rapid tumor cell growth by supplying cells with metabolic building blocks, which also serve as growth promoting signaling molecules [18]. Due to the major role of glycolysis in cancer, one would expect that inhibiting glycolysis would be a powerful strategy in cancer treatment. Unfortunately, clinical trials testing 2DG did not provide patients with the desired benefit [19]. Here, we found that compound 6 dramatically increased the efficacy of 2DG in culture, suggesting that it interferes with metabolic pathways circumventing glycolysis inhibition. Furthermore, Khan et al. showed that biguanides, including metformin, which inhibit mitochondrial activity, synergize with glycolysis inhibitors to kill tumor cells [20]. This raises the possibility that by inhibiting mitochondrial activity compound 6 sensitizes cells to 2DG.

Pusapati et al. showed that mTOR inhibition supports survival of tumor cells treated with glycolysis inhibitors [21]. We found that compound 6 treatment leads to mTOR inhibition in glucose-starved cells. Because compound 6 inhibits mTOR only upon glucose starvation, we argue that it is not likely to function as a direct mTOR inhibitor. Nevertheless, mTOR inhibition under metabolic stress is an expected cellular response to energetic crisis [10], further supporting the conclusion that compound 6 enhances the energetic stress in glucose-starved cells.

Our previous study identified compounds which inhibit mitochondrial membrane potential, inhibit mTOR, and kill tumor cells upon glucose starvation in culture [13]. Importantly, we found that these compounds synergize with a vascular endothelial growth factor (VEGF) inhibitor in vivo. Here, we found a new compound, compound 6, with similar activity in culture, which may synergize with VEGF inhibitors in vivo as well. Interestingly, we found that compound 6 enhances the toxicity of 2DG in culture, raising the possibility that it might do so in vivo as well. We therefore conclude that compound 6 is not toxic in cells growing in standard cell culture conditions, and may hold therapeutic promise when applied in combination with drugs that increase metabolic stress in tumors such as VEGF inhibitors or 2DG. 

## 4. Materials and Methods

### 4.1. Amuvatinib and Its Derivatives

Amuvatinib (MP-470) was purchased from A2S (A4237-5, Saint Jean d’Illac, France). Compounds 1–7 were synthesized by chem-space.com (Riga, Latvia). Compounds 8–12 were synthesized by enamine.net (Riga, Latvia).

### 4.2. Cell cultures and Glucose Starvation

All cell lines were obtained from the ATCC and cultured in Dulbecco’s Modified Eagle’s Medium (DMEM) (Biological Industries, Rechovot, Israel) containing Antibiotic-Antimycotic (Tivan Biotech, Rechovot, Israel) 1 mM sodium pyruvate (Biological Industries, Rechovot, Israel), and 10% fetal bovine serum (FBS) (Biological Industries, Rechovot, Israel). Cells were maintained at 37 °C and 5% CO_2_. Glucose starvation: medium was changed to glucose-free DMEM containing Antibiotic-Antimitotic and 10% FBS. All compounds in the screen were diluted in DMSO. 

### 4.3. Cell Viability Assays

Cell viability was measured using Crystal Violet staining. Cells were plated in 24-well plates with treatments of glucose-depleted medium and indicated compounds. Cells were then incubated at 37 °C, 5% CO_2_ for 48 h, followed by aspirating the medium and washing with Phosphate buffered saline ×1 (PBS×1) (Biological Industries). Next, 250 µL of Crystal Violet staining solution (0.5% (w/v) Crystal Violet powder, 20% methanol) was added to plates. Plates remained at room temperature for 10 min, followed by discarding the Crystal Violet staining solution and washing 5 times with distilled water. Plates were dried and 250 µL of 10% acetic acid was added to each well. Absorbance was then read at wavelength 570 nm.

### 4.4. Cell Death Assay

Cell death was determined using propidium iodide (PI) staining and FACS. Sixteen hours post-treatment, the medium, including floating cells, was collected into the collection tube. Attached cells were washed with PBS, which was also collected into the collection tube. To detach the cells, 1 mL of Trypsin was then added and cells were incubated for 3 min at 37 °C. Next, 4 mL medium was added to the trypsin and all were collected into the collection tube. The collection tube was centrifuged for 5 min in 1200 rpm, followed by aspirating the medium and two PBS washes. Cells were suspended with 1 mL of PBS and 1 μL of propidium iodide (PI) (Sigma-Aldrich, Dimethyl sulfoxide) was added adjacent to analysis by flow cytometer (Sysmex, Kobe, Japan) equipped with FCS Express software according to manufacturer’s instructions (Kobe, Japan).

### 4.5. Mitochondrial Membrane Potential

Mitochondrial membrane potential was measured using FACS and TMRE (tetramethylrhodamine, ethyl ester) mitochondrial membrane potential assay kit (Cayman Chemical, Ann Arbor, MI, USA). Cells were plated in 6-well plates and treated with and without glucose and 20 µM compound 6 or vehicle (DMSO). TMRE was added to the media to a final concentration of 500 nM and incubated for 20 min at 37 °C. Cells were washed with PBS and harvested using 200 µL of trypsin. Cells were collected with 800 µL of 0.2% BSA Containing PBS and fluorescence was measured using FACS.

### 4.6. Lysis and Samples Preparation

Cell lysis was performed on ice. Cells were washed one time with PBS and scraped with radioimmunoprecipitation assay (RIPA) lysis buffer (150 mM NaCl; 50 mM Tris pH = 8.0; 1% Triton X-100; 0.5% Sodium deoxycholate; 0.1% SDS). Sample was then sonicated and centrifuged at 4 °C for 20 min. The supernatant was collected and stored in −20 °C. Protein concentrations were determined using Pierce™ BCA Protein Assay Kit (Thermo Scientific, Waltham, MA, USA).

### 4.7. Gel Electrophoresis and Immunoblotting

Lysates were mixed with sample loading buffer 5× (250 mM Tris·HCl pH 6.8; 10% SDS; 30% Glycerol; 10 mM DTT; 0.05% (w/v) Bromophenol Blue), followed by 10 min incubation at 96 °C and spin-down. Samples were then loaded to SDS-PAGE followed by transferring to nitrocellulose membrane. Membranes were then blocked by soaking in milk (5% skim milk; Tris buffer saline ×1 contains 0.05% TWEEN20 (TBST)) for 1 h. Membranes were then covered with primary antibody solution for 1 h in room temperature or overnight in 4 °C, followed by washing with TBST and incubation with secondary antibodies for 1 h at room temperature. Images were developed using Western blot Serius horseradish peroxidase (HRP) substrate kit and an electrochemiluminescence imager (Thermo Scientific,) and processed using GIMP 2.8 software. 

### 4.8. Antibodies

HSC70 antibody (sc-7298) was obtained from Santa Cruz (Dallas, TX, USA). Antibodies for Phospho-p70 S6 Kinase (Thr389) (9206s), Total p70 S6 Kinase (2708s), 4E-BP1 (C-9644S), P-S6 Ribosomal Protein (Ser240/244) (2215S), P-4E-BP1 (Ser65) (9451S), Anti-Rabbit (C-7074S) and Anti-Mouse (C-7076S) were obtained from Cell Signaling (Danvers, MA, USA).

### 4.9. Statistics

Means ± SEM of results are obtained from at least three independent experiments. Statistical analyses were done using GraphPad Prism 8.2.1 software (San Diego, CA, USA). P-values were calculated by using *t*-test or ANOVA and were considered significant if *P* ≤ 0.05.

## Abbreviations

2DG	2-deoxy-glucose
mTOR	mammalian target of rapamycin
AMPK	AMP activated protein kinase
FACS	fluorescence-activated cell sorting
TMRE	tetramethylrhodamine, ethyl ester
PI	propidium iodide
DMSO	dimethyl sulfoxide
